# Supplementary material for: Temporal clusters of age-related behavioral alterations captured in smartphone touchscreen interactions
Source: iScience. 2022 Aug 5;25(8):104791. doi: 10.1016/j.isci.2022.104791 (PMC9418599; doi:10.1016/j.isci.2022.104791)
Supplement: Document S1. Figures S1–S16 [file mmc1.pdf]

iScience, Volume *25*

Supplemental information

Temporal clusters of age-related behavioral alterations captured in smartphone touchscreen interactions

Enea Ceolini, Ruchella Kock, Guido P.H. Band, Gijsbert Stoet, and Arko Ghosh

Temporal clusters of age-related behavioral alterations captured in smartphone touchscreen interactions

July 11, 2022

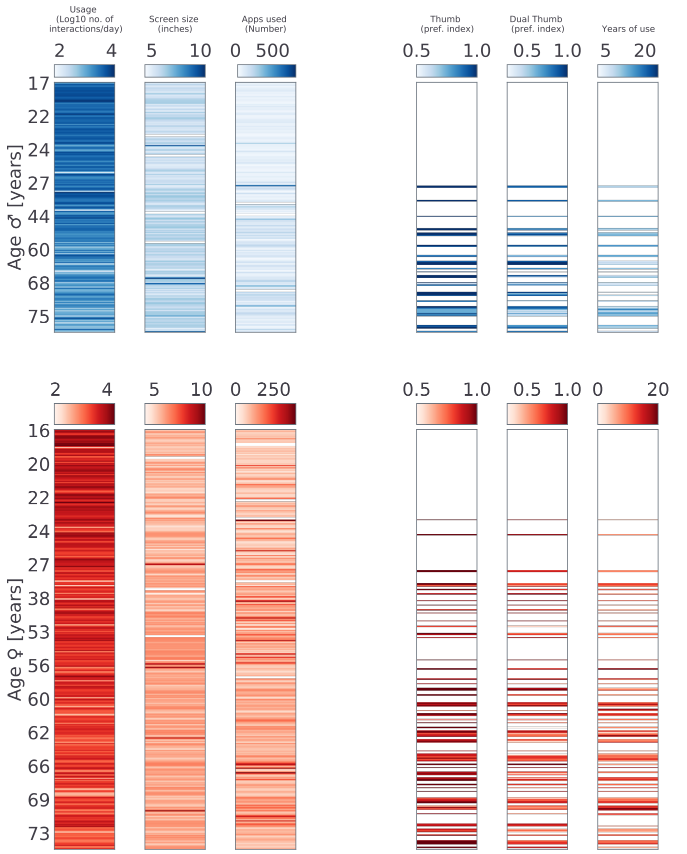

Figure S1: *Overview of smartphone-related parameters.* The data is sorted by gender and age. White spaces indicate unavailable data. The first three columns were derived using objective measures, whereas the later three columns are based on self-reports, related to Figure 1.

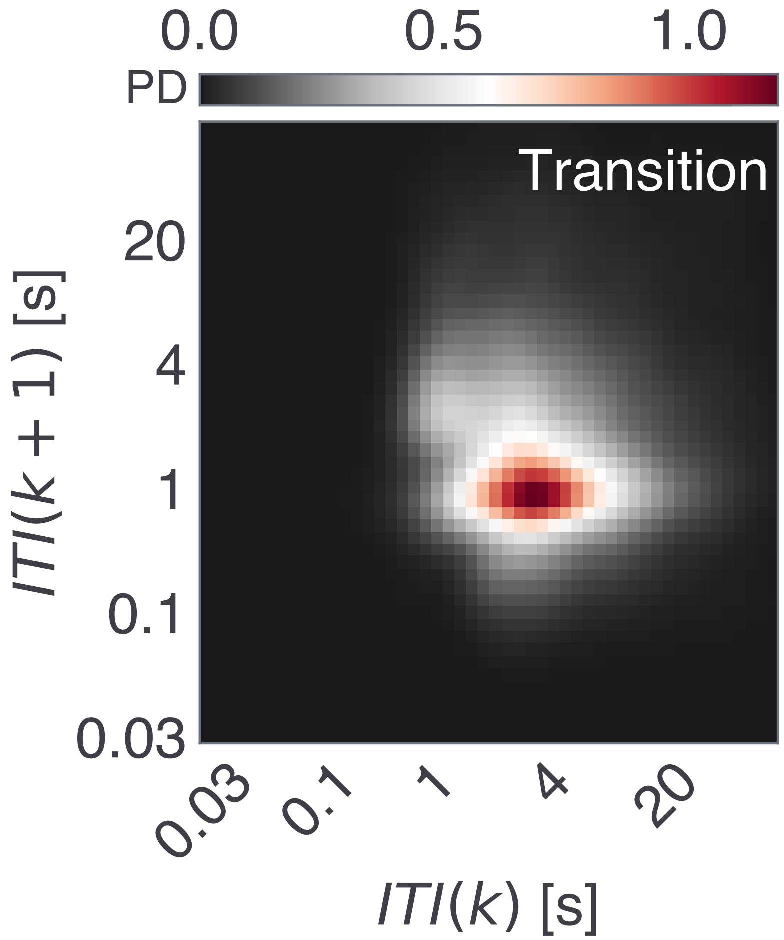

Figure S2: *The Transition JID.* Population means of the probability densities derived at each two-dimensional bin, related to Figure 1.

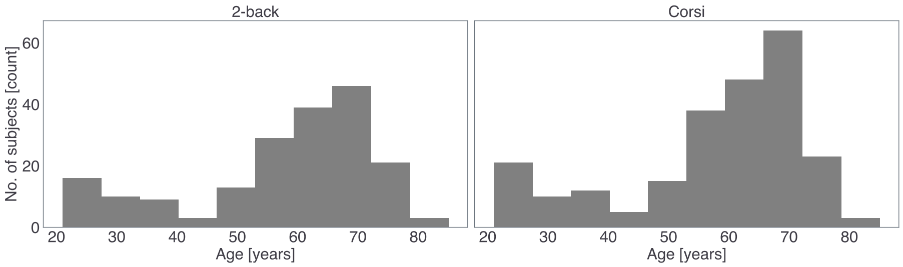

Figure S3: *The distribution of age of the participants who performed the Corsi block and 2-back tasks.* The distribution of age of the participants who performed the Corsi block and 2-back tasks, related to Figure 2.

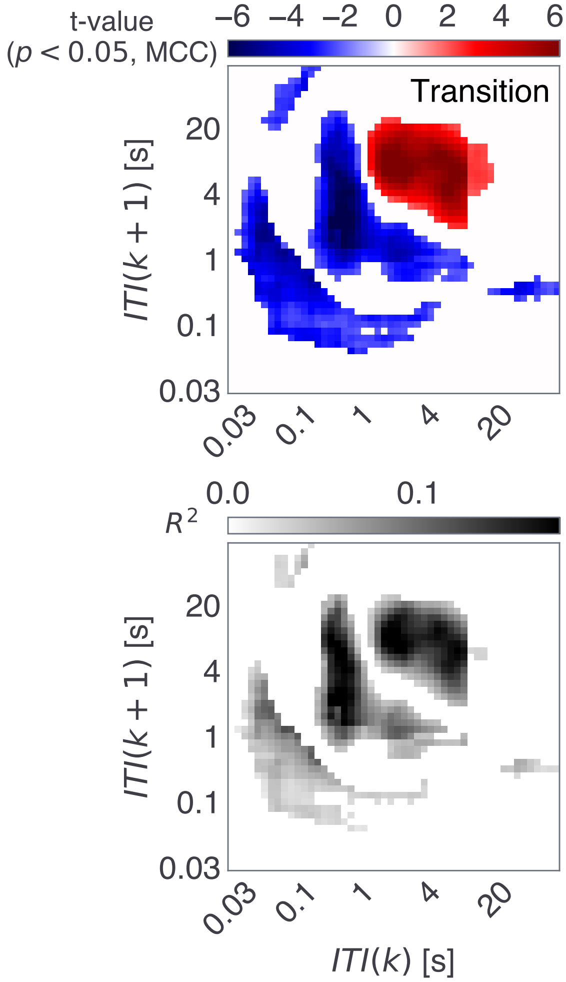

Figure S4: *Transition JID correlates with the choice reaction time.* The t-statistics correspond to the choice reaction time (red-blue image) and the corresponding R^2 of the full regression model (incl. gender, gray image). The statistics were corrected for multiple comparisons using two-dimensional clustering, related to Figure 2.

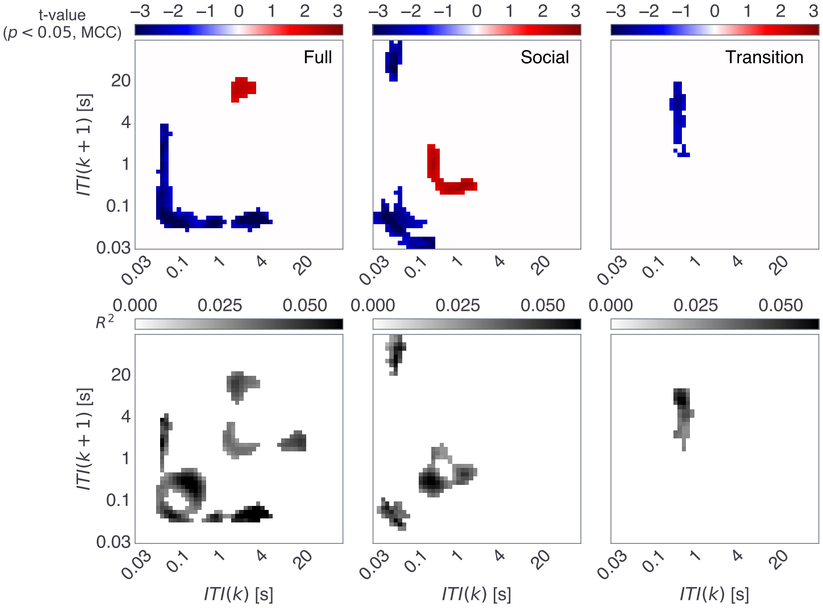

Figure S5: *Smartphone correlates of simple reaction time.* The smartphone behavioral correlates of simple reaction time on the Full, Social, and Transition JIDs. The t-statistics correspond to the cognitive test (red-blue image) and the corresponding R^2 of the full regression model (incl. gender, gray image). The statistics were corrected for multiple comparisons using two-dimensional clustering, related to Figure 2.

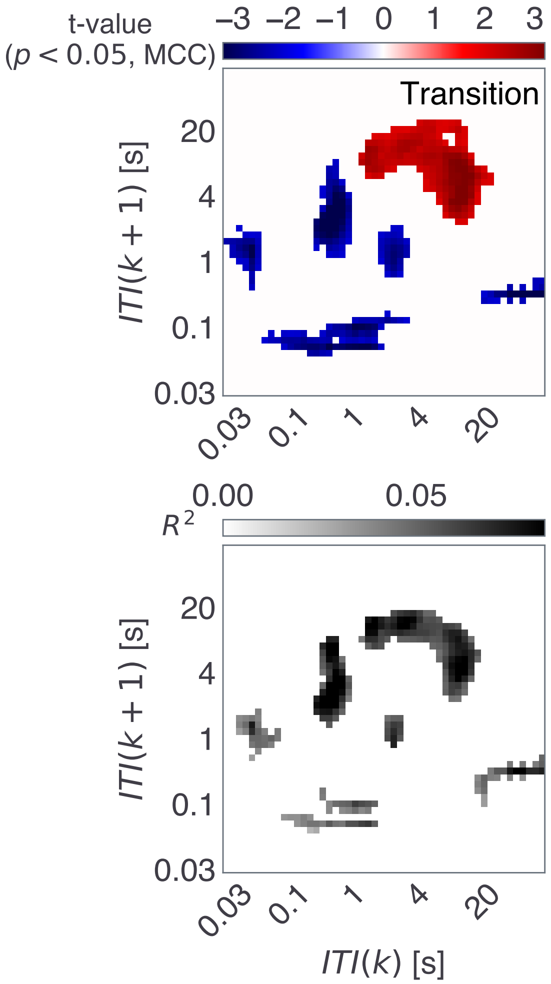

Figure S6: *Transition JID correlates of global cost.* The smartphone behavioral correlates of simple reaction time on the Full, Social, and Transition JIDs. The t-statistics correspond to the cognitive test (red-blue image) and the corresponding R^2 of the full regression model (incl. gender, gray image). The statistics were corrected for multiple comparisons using two-dimensional clustering, related to Figure 2.

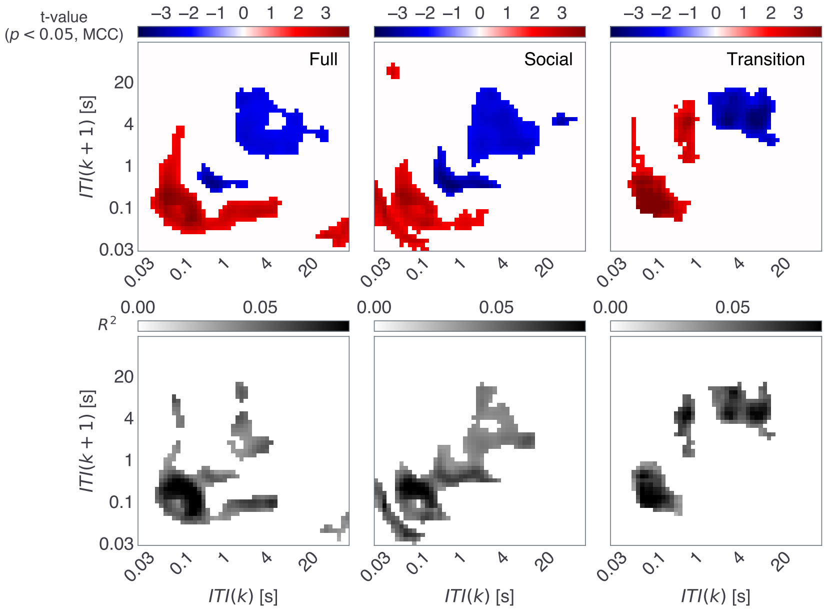

Figure S7: *Smartphone behavioral correlates of the 2-back test.* The smartphone behavioral correlates of 2-back D' on the Full, Social, and Transition JID. The t-statistics correspond to the cognitive test (red-blue image) and the corresponding R^2 of the full regression model (incl. gender, gray image). The statistics were corrected for multiple comparisons using two-dimensional clustering, related to Figure 2.

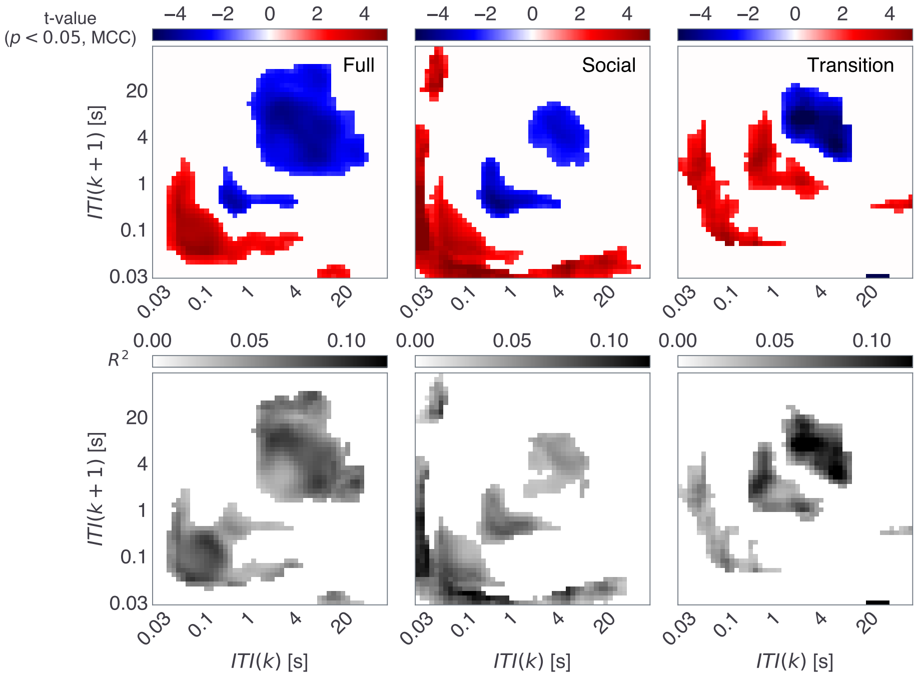

Figure S8: *Smartphone correlates of Corsi Block.* The smartphone behavioral correlates of the span on the Corsi Block task on the Full, Social, and Transition JID. The t-statistics correspond to the cognitive test (red-blue image) and the corresponding R^2 of the full regression model (incl. gender, gray image). The statistics were corrected for multiple comparisons using two-dimensional clustering, related to Figure 2.

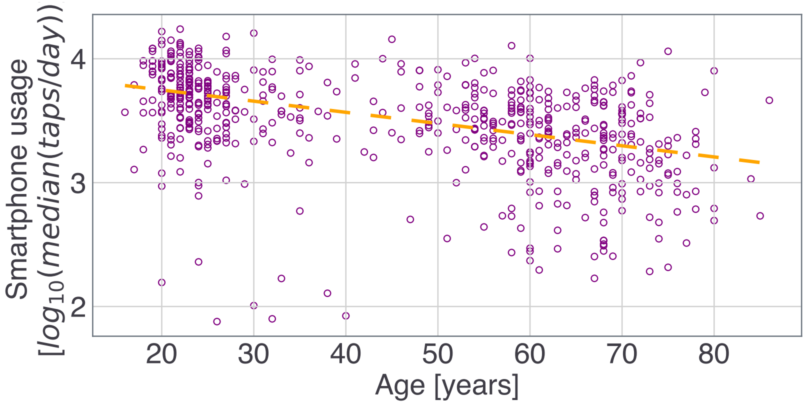

Figure S9: *Smartphone correlates of age.* The amount of smartphone usage correlates with age. Adjusted response plot showing the linear relation between smartphone usage vs. age. Based on robust linear regression against age and gender, related to Figure 3.

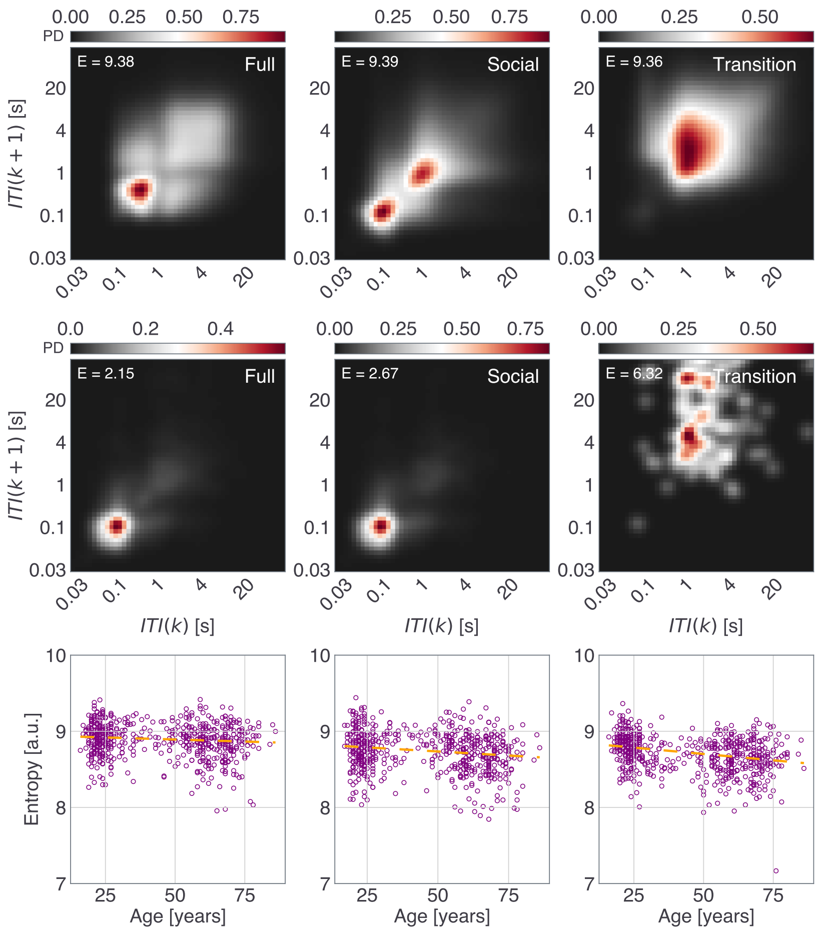

Figure S10: *Smartphone entropy.* The entropy of the JID is related to age. The Full, Social, and Transition JIDs with min and max entropy values in the sampled population. The adjusted response plots show the linear relation between the corresponding entropy vs. age. Based on robust linear regression against age and gender, related to Figure 3.

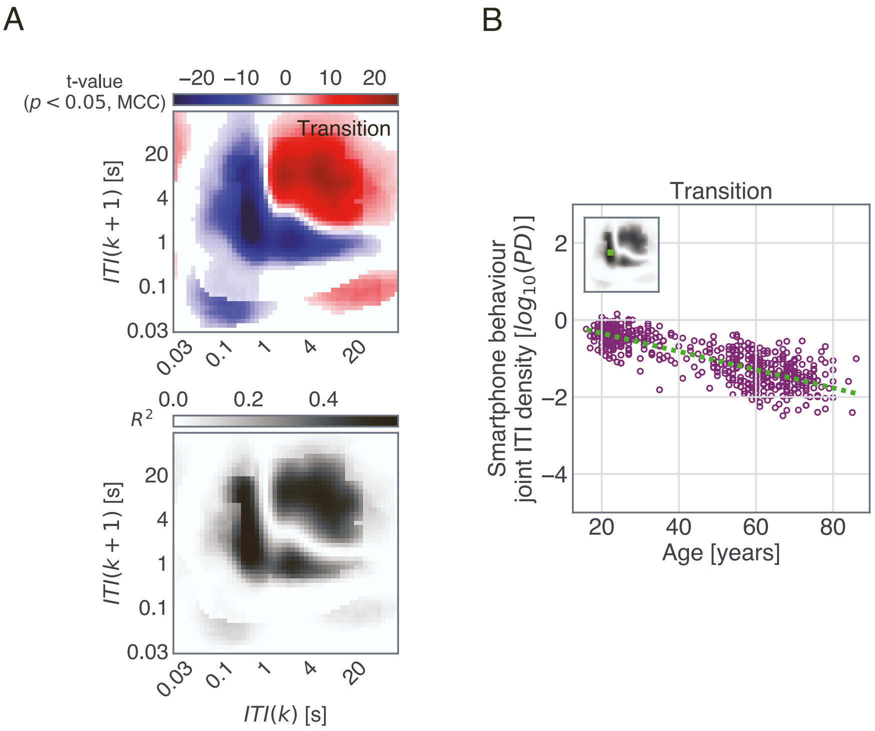

Figure S11: *Transition JID correlates of age.* The correlation between Transition JID and age. Legend same as in Fig. 3 but for Transition JID, related to Figure 3.

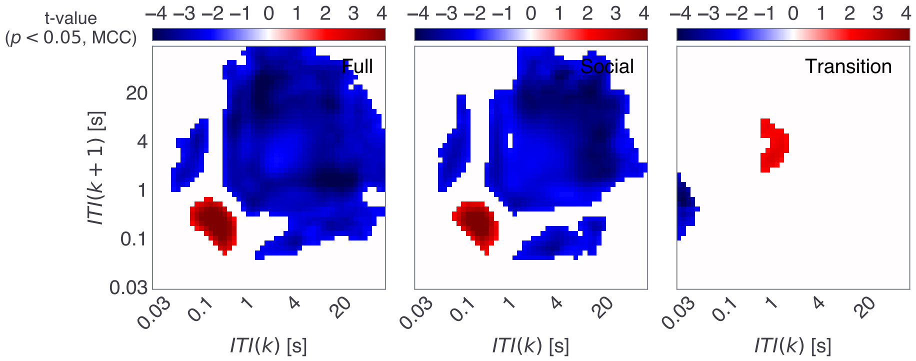

Figure S12: *Smartphone correlates of gender on the JIDs.* The t-statistics (red-blue images) for the variable gender (dummy variable in the regression model including age). Male 1, and female 2 were used as dummy variables. Note, females (2) showed higher probability densities of short consecutive intervals, related to Figure 3.

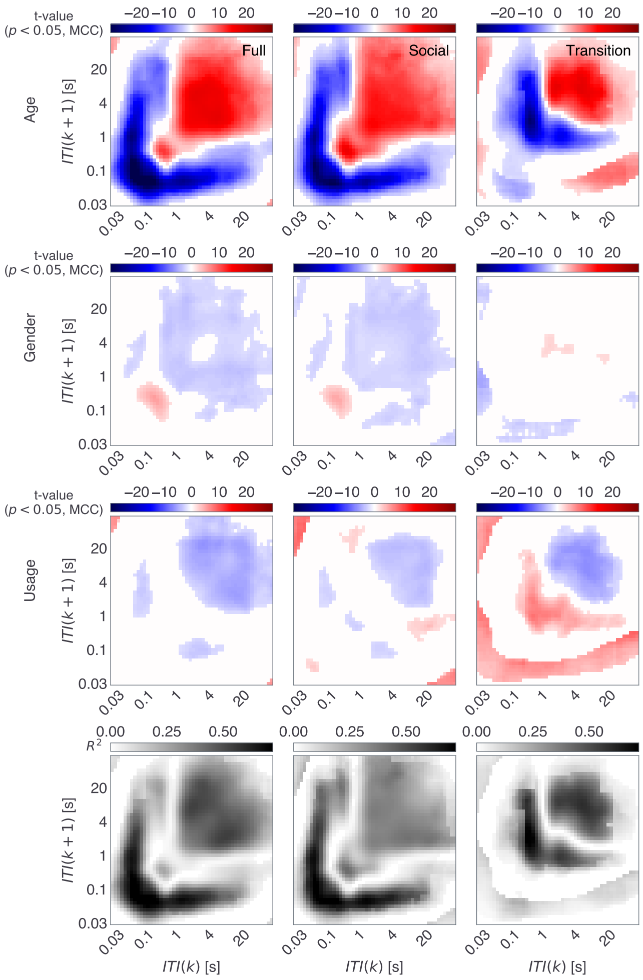

Figure S13: *A multivariate model including age, gender, and smartphone usage linking to the JIDs.* The results of mass univariate regression include the amount of smartphone usage in terms of the median number of interactions per day in addition to age and gender in the mass regressions conducted at each two-dimensional bin. The t-statistics for all of the variables and corresponding R^2 of the full model. All statistics were corrected for multiple comparisons using two-dimensional clustering $\alpha = 0.05$, related to Figure 3.

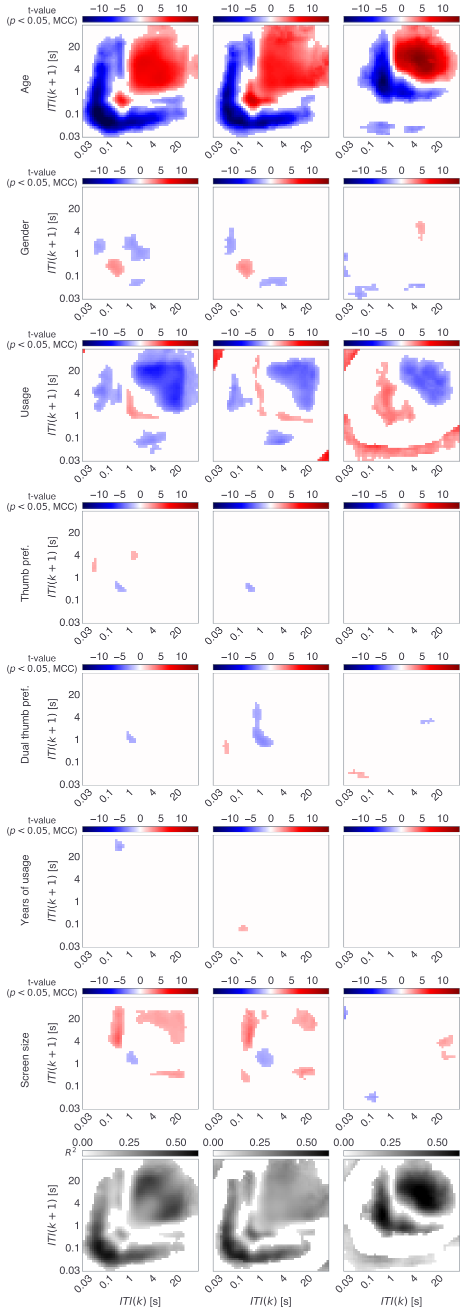

Figure S14: *Age-related correlates in JIDs remain in the presence of other smartphone use-related variables.* We used a multivariate model to explain the inter-individual differences in the JID and this model included age, gender, smartphone usage, thumb preference score, dual thumb preference score, and the years of the smartphone experience. The t-statistics for all of the variables and corresponding R^2 of the full model. All statistics were corrected for multiple comparisons using two-dimensional clustering $\alpha = 0.05$, related to Figure 3.

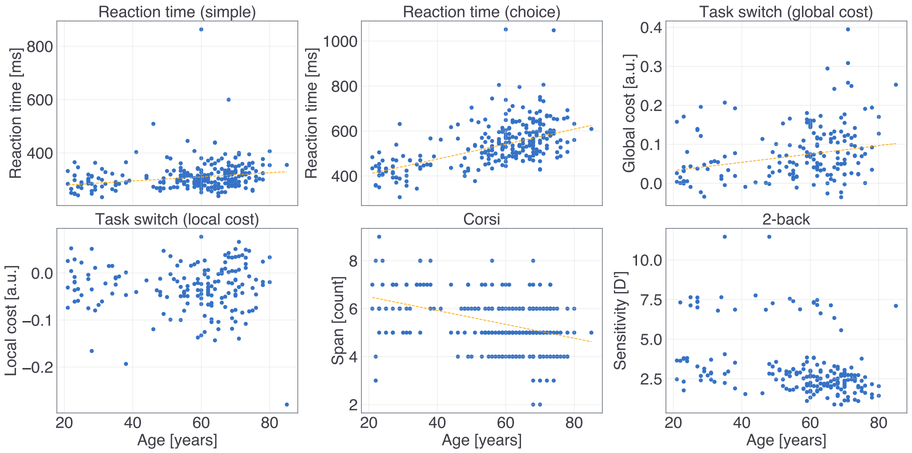

Figure S15: *Age-related correlates of cognitive tests.* The adjusted response plots are based on multivariate regression models containing the variables age and gender. Regression fits with $p < 0.05$ for the variable age are plotted, related to Figure 3.

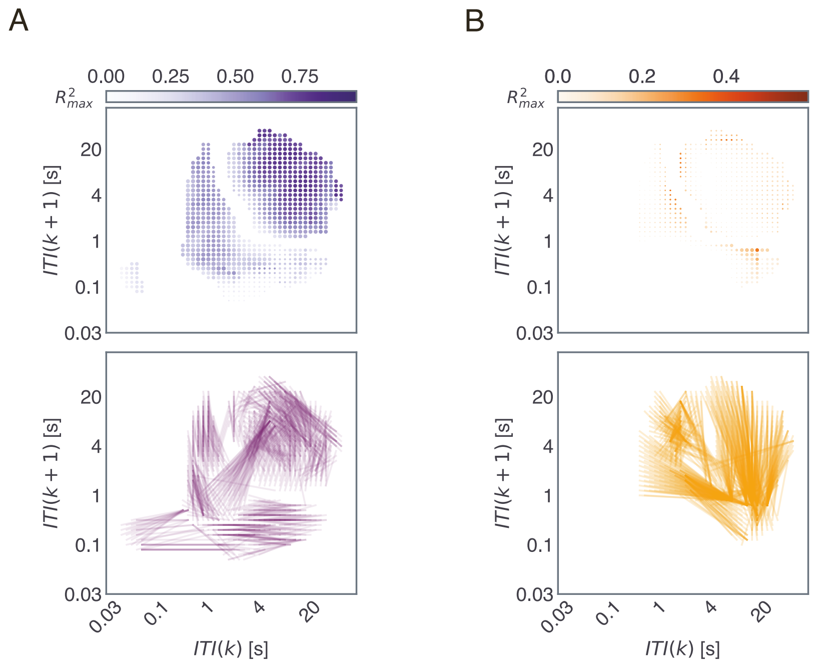

Figure S16: *Accelerated & decelerated aging captured on Transition JID.* (A) Consistent relationships and the corresponding links and (B) inconsistent relationships and the corresponding links. Legend same as in Figure 4 C & D, related to Figure 4.
